# Influence of Fluoride-Resistant *Streptococcus mutans* Within Antagonistic Dual-Species Biofilms Under Fluoride *In Vitro*

**DOI:** 10.3389/fcimb.2022.801569

**Published:** 2022-02-28

**Authors:** Keke Zhang, Yangfan Xiang, Youjian Peng, Fengyu Tang, Yanfan Cao, Zhenjie Xing, Yejian Li, Xiangyan Liao, Yan Sun, Yan He, Qingsong Ye

**Affiliations:** ^1^ School and Hospital of Stomatology, Wenzhou Medical University, Wenzhou, China; ^2^ Center of Regenerative Medicine, Renmin Hospital of Wuhan University, Wuhan, China; ^3^ Tianyou Hospital, Wuhan University of Science and Technology, Wuhan, China

**Keywords:** fluoride-resistant *Streptococcus mutans*, *Streptococcus sanguinis*, antagonistic dual-species biofilm, biofilm homeostasis, cariogenic virulence, dental caries

## Abstract

The widespread application of fluoride, an extremely effective caries prevention agent, induces the generation of fluoride-resistant strains of opportunistic cariogenic bacteria such as fluoride-resistant *Streptococcus mutans* (*S. mutans*). However, the influence of this fluoride-resistant strain on oral microecological homeostasis under fluoride remains unknown. In this study, an antagonistic dual-species biofilm model composed of *S. mutans* and *Streptococcus sanguinis* (*S. sanguinis*) was used to investigate the influence of fluoride-resistant *S. mutans* on dual-species biofilm formation and pre-formed biofilms under fluoride to further elucidate whether fluoride-resistant strains would influence the anti-caries effect of fluoride from the point of biofilm control. The ratio of bacteria within dual-species biofilms was investigated using quantitative real-time PCR and fluorescence *in situ* hybridization. Cristal violet staining, scanning electron microscopy imaging, and 3-(4,5-dimethylthiazol-2-yl)-2,5-diphenyl-2H-tetrazolium bromide assay were used to evaluate biofilm biomass, biofilm structure, and metabolic activity, respectively. Biofilm acidogenicity was determined using lactic acid and pH measurements. The anthrone method and exopolysaccharide (EPS) staining were used to study the EPS production of biofilms. We found that, in biofilm formation, fluoride-resistant *S. mutans* occupied an overwhelming advantage in dual-species biofilms under fluoride, thus showing more biofilm biomass, more robust biofilm structure, and stronger metabolic activity (except for 0.275 g/L sodium fluoride [NaF]), EPS production, and acidogenicity within dual-species biofilms. However, in pre-formed biofilms, the advantage of fluoride-resistant *S. mutans* could not be fully highlighted for biofilm formation. Therefore, fluoride-resistant *S. mutans* could influence the anti-caries effect of fluoride on antagonistic dual-species biofilm formation while being heavily discounted in pre-formed biofilms from the perspective of biofilm control.

## Introduction

Caries is a disease of chronic and progressive destruction of the hard tissue of teeth caused by multiple factors, including bacteria (Mathur and Dhillon, 2018). In the United States, for example, caries prevalence indicated that about 45.8% of children aged 2–19 years old had experienced dental caries in primary and permanent dentitions, while 57% of adults had experienced dental caries (Fleming and Afful, 2018; Brandfass et al., 2019). With the growing sugar intake becoming a global issue, the incidence of dental caries has increased rapidly and has a profound impact on the general health of individuals (Van Loveren, 2019). Over 1,000 different microbial species, also known as oral biofilms, have been identified within the dental plaque (Dewhirst et al., 2010). The caries ecological hypothesis proposed by Phil D. Marsh suggested that there was a balance in the microecology of oral plaque (Marsh et al., 2015). Dental caries result from the disruption of homeostasis in this microecology. Excessive sugar intake and reduced salivary production contribute to the decrease in pH in oral biofilms. Consecutively, acid-tolerant and acid-producing bacteria would survive and strengthen acid production, thus causing the occurrence of demineralization and the development of caries (Marsh et al., 2015).

Fluoride, such as sodium fluoride (NaF), acidulated fluorophosphates (APF), and stannous fluoride (SnF_2_), is commonly used to prevent the development of caries. They are used in clinical anti-caries applications, including toothpaste, mouthwash, gel, varnishes, and tooth-filling materials that release ionic fluoride (Anusavice et al., 2005; Pitts et al., 2017). Fluoride enhances the acid resistance of teeth by inhibiting demineralization and enhancing remineralization and also inhibits the growth and metabolism of bacteria by suppressing the activities of enzymes such as enolase and ATPase (Ten Cate, 2004; Oh et al., 2017). When the pH of the extracellular environment decreases, protons (H^+^) and fluoride (F^+^) from fluoride materials diffuse into bacterial cells and exist as hydrogen fluoride (HF) in the cytoplasm (Marquis et al., 2003). This influx of HF both directly and indirectly affects the growth and cariogenicity of bacteria (Liao et al., 2017). The protective effect of fluoride on the enamel was observed at 0.02 mg/L fluoride, and fluoride significantly reduced the number of *Streptococcus mutans* (*S. mutans*) at a concentration starting from 0.25 g/L. *S. mutans* is one of the main opportunistic cariogenic bacteria, and its virulence factors are acid production, acid tolerance, and adhesion (Liu et al., 2015).

However, the widespread application of fluoride induces the generation of fluoride-resistant strains, including opportunistic cariogenic bacteria such as fluoride-resistant *S. mutans*. As early as 1980, transient fluoride-resistant *S. mutans* strains were isolated from the plaque of radiation-induced xerostomia patients who were treated daily with preventive NaF gel (Streckfuss et al., 1980). Laboratory-induced and characteristically stable (at least 50 generations) fluoride-resistant *S. mutans* were used to study their phenotypes and fluoride-resistant mechanisms (Liao et al., 2017). This type of fluoride-resistant *S. mutans* is generally able to withstand fluoride concentrations three times higher than its wild strains (Liao et al., 2017). Some studies have reported the phenotypic characteristics of fluoride-resistant strains, such as the stability of fluoride resistance, fitness, growth, acidogenicity, and cariogenicity. These results showed that there are many significant differences in these phenotypic characteristics between the fluoride-resistant strains and their wild strains, such as higher fluoride resistance, higher acid tolerance, lower growth, and some controversial cariogenitic characteristics (Van Loveren et al., 1991; Zhu et al., 2012; Liao et al., 2015; Cai et al., 2017; Liao et al., 2017; Liao et al., 2018; Lee et al., 2021). These genetic stability differences could be caused by genetic mutations, as revealed by gene sequencing (Liao et al., 2015; Liao et al., 2018, Lee et al., 2021). Although the complete mechanism of *S. mutans* fluoride resistance needs to be further studied, some genes or genetic loci have been found to be responsible for the fluoride resistance of *S. mutans* (Liao et al., 2016; Men et al., 2016; Murata and Hanada, 2016; Tang et al., 2019; Lu et al., 2020; Yu et al., 2020). However, the ecological effects of fluoride-resistant *S. mutans* remain unknown.

Accumulating evidence indicates that there is a competitive and antagonistic relationship between *S. mutans* and *S. sanguinis* (Marsh and Zaura, 2017). A significant association has been reported between the *S. mutans* and *S. sanguinis* ratio and severe early childhood caries in dental plaque (Mitrakul et al., 2016). The presence of *S. sanguinis* had a negative relationship with the occurrence of dental caries. Kreth et al. reported that *S. sanguinis* could produce hydrogen peroxide to inhibit *S. mutans* (Kreth et al., 2008). *S. mutans* can suppress the adhesion of *S. sanguinis* through mutacin production (Valdebenito et al., 2018). The balance between these two strains represents the equilibrium of dental plaque to some extent (Sun et al., 2019; Du et al., 2021). However, there have been no studies on the influence of fluoride-resistant *S. mutans* on oral microecological homeostasis under fluoride. The present study used an antagonistic dual-species biofilm model composed of *S. mutans* and *S. sanguinis* to investigate the influence of fluoride-resistant *S. mutans* on microbial flora under fluoride. We hypothesized that, under the screening effect of fluoride, fluoride-resistant *S. mutans* might gain a survival advantage within antagonistic dual-species biofilms, which destroys the ecological balance of oral biofilms and leads to the occurrence and development of dental caries. Eventually, it would influence the anti-caries effect of fluoride. This *in vitro* study was designed to verify this hypothesis.

## Materials and Methods

### Bacterial Strains and Growth Conditions

*S. mutans* UA159 and *S. sanguinis* ATCC 10556 were obtained from the School and Hospital of Stomatology, Wenzhou Medical University. Fluoride-resistant *S. mutans* was induced *in vitro* as previously described, with modifications (Zhu et al., 2012). Briefly, an overnight bacterial suspension was inoculated on a brain heart infusion (BHI, Oxoid, Basingstoke, UK) agar plate containing 0.5 g/L NaF for 48 h growth, where a single colony of *S. mutans* was picked and passaged on BHI agar without NaF for 50 generations. The fluoride-resistant characteristics of *S. mutans* were confirmed on BHI solid medium with 0.5 g/L NaF. BHI medium was used for bacterial amplification, and BHI with 1% sucrose (BHIS) was used for biofilm formation. The growth conditions were 37°C and 5% CO_2_.

### Biofilm Culture

In this study, we characterized fluoride-resistant *S. mutans* in biofilm formation and in pre-formed biofilms under fluoride. For biofilm formation, overnight bacterial suspensions of one or two species were diluted 50-fold into BHIS containing 0, 0.275, and 1.25 g/L NaF (0.275 and 1.25 g/L NaF were the fluoride content in regular and prescription toothpaste, respectively, after 3-fold dilution) and incubated for 24 h (Nassar and Gregory, 2017). For the pre-formed biofilm assay, after 24 h of biofilm formation without fluoride, the culture medium was replaced with fresh BHIS with different concentrations of NaF and incubated for another 24 h.

The groups in this experiment were divided into single-species biofilms of *S. mutans* wild-type strain (*S.m* WT), single-species biofilms of fluoride-resistant *S. mutans* (*S.m* FR), single-species biofilms of *S. sanguinis* (*S.s*), dual-species biofilms of the wild type of *S. mutans* strain and *S. sanguinis* (*S.m* WT + *S.s*), and dual-species biofilms of fluoride-resistant *S. mutans* and *S. sanguinis* (*S.m* FR + *S.s*). Each group was treated with different fluoride concentrations, which included control, low (0.275 g/L), and high concentrations (1.25 g/L).

### Crystal Violet Staining

The biomass of the biofilm was determined using crystal violet (CV) staining (Zhu et al., 2021). Biofilms in 96-well plates were washed with phosphate-buffered saline (PBS) and fixed with methanol for 15 min. Air-dried biofilms were stained with 100 µl of 0.1% crystal violet solution for 30 min and washed with PBS. Images of the stained biofilms were captured using a stereo microscope (Nikon SMZ800, Nikon Corporation, Japan). Next, they were dissolved in 200 µl of 33% acetic acid with shaking for 15 min, and the absorbance was measured at 590 nm using a microplate reader (SpectraMax M5, Molecular Devices, USA).

### Metabolic Activity

For metabolic activity assessment, biofilms growing on round glass wafers were washed with PBS to remove planktonic bacteria and stained with 1 ml 0.5% 3-(4,5-dimethylthiazol-2-yl)-2,5-diphenyl-2H-tetrazolium bromide (MTT) solution (dissolved in PBS) for 1 h. Subsequently, the wafers were transferred to a new plate with dimethyl sulfoxide (1 ml per well). Thereafter, the plate was shaken for 30 min to completely dissolve the crystals. A 200-µl aliquot of the solution was measured at 540 nm using a microplate reader (SpectraMax M5, Molecular Devices, USA).

### Lactic Acid and pH Measurement

Lactic acid and pH measurements were conducted to monitor acid production (Sun et al., 2019). Biofilms in wafers were first washed with cysteine peptone water and then cultured in buffered peptone water (BPW) containing 0.2% sucrose (1 ml/well) for 3 h to allow acid production. Lactic dehydrogenase was used to quantify lactate concentrations in the BPW solution. The absorbance was read at 340 nm, and standard curves were generated using a lactic acid standard.

For pH measurement, the supernatant of the biofilms was measured using a pH meter (Mettler Toledo Instruments Co., Ltd., Shanghai, China).

### Scanning Electron Microscopy Imaging

SEM imaging was performed to observe the morphology and structure of the biofilms (Liu et al., 2013). Biofilms were fixed with 2.5% glutaraldehyde and dehydrated using an ethanol gradient (50, 60, 70, 80, 90, and 95% and absolute ethyl alcohol) for 30 min at each concentration. Dry biofilms were sputter-coated with gold–palladium for observation using SEM at ×2,000 magnification (Hitachi, Tokyo, Japan).

### Water-Insoluble Exopolysaccharide Measurement

The water-insoluble EPS of biofilms was measured using the anthrone method (Sun et al., 2021). Briefly, the biofilms were collected, washed twice with sterile water, and resuspended in 0.4 M NaOH. After centrifugation, 200 µl of the suspension was mixed with 600 µl of anthrone reagent and incubated at 95°C for 6 min. The absorbance was monitored at 625 nm using a microplate reader (SpectraMax M5, Molecular Devices, USA). Standard curves were prepared using dextran standard.

### Confocal Laser Scanning Microscopy Assay

To observe the EPS production in biofilms, fluorescence staining was conducted (Liu et al., 2013). Alexa Fluor-647 dextran conjugate (Molecular Probes, Invitrogen Corp., Carlsbad, CA, USA) was added to the culture medium at the beginning of biofilm formation to label the EPSs. At the end of biofilm formation, the biofilms were stained with SYTO 9 (Molecular Probes, Invitrogen Corp., Carlsbad, CA, USA) for total bacteria measurement. Random fields were selected, and images were captured using a ×60 oil immersion lens with a confocal laser scanning microscope (Nikon Corporation, Tokyo, Japan).

### Quantitative Real-time PCR Assay

To determine the ratio of *S. mutans* and *S. sanguinis* in dual-species biofilms, TB Green Premix Ex Taq™ II kit (Takara Bio Inc., Otsu, Japan) was used for qRT-PCR analysis. The total DNA of dual-species biofilms was extracted using Rapid Bacterial Genomic DNA Isolation Kit (Sangon Biotech, Shanghai, China). The primers used in this study were the same as those previously described (Huang et al., 2015) (**Supplementary Table 1**). A total of 20 µl of reaction mixture contained 10.0 µl 2× TB Green Premix Ex Taq II, 0.8 µl forward primer, 0.8 µl reverse primer, 2.0 µl cDNA, and 6.4 µl sterilized distilled water. We used a LightCycler 96 instrument (Roche Diagnostics, Basel, Switzerland) and programmed the system for 30 s of pre-denaturation at 95°C, followed by 40 cycles of 5-s denaturation at 95°C, 30 s annealing at 55°C, and 30-s extension at 72°C. The standard curves of *S. mutans* and *S. sanguinis* were generated based on the known quantities of bacteria by CFU count.

### Fluorescence *In Situ* Hybridization

FISH was used to observe the proportion of bacterial components in the dual-species biofilms. Briefly, after washing with PBS twice, the biofilms on the wafers were fixed with 4% paraformaldehyde for 6 h. Lysozyme was used to lyse the cell wall. The biofilms were then dehydrated with gradient ethanol and dried at 46°C for 10 min. Specific fluorescent probes (**Supplementary Table 2**) were used to stain *S. mutans* and *S. sanguinis* within dual-species biofilms (Zheng et al., 2013). A confocal laser scanning microscope (Nikon Corporation, Tokyo, Japan) was used to capture the FISH results using a ×60 oil immersion lens.

### Statistical Analysis

All experiments were repeated independently at least thrice. One-way analysis of variance was performed, and statistical significance was set at *p <*0.05 using SPSS software (version 24.0; SPSS Inc., Chicago, IL, USA).

## Results

### Fluoride-Resistant *S. mutans* Obtained Remarkable Competitive Advantage Within Dual-Species Biofilms During Biofilm Formation While Not in Pre-Formed Biofilms Under NaF

**Figure 1:**
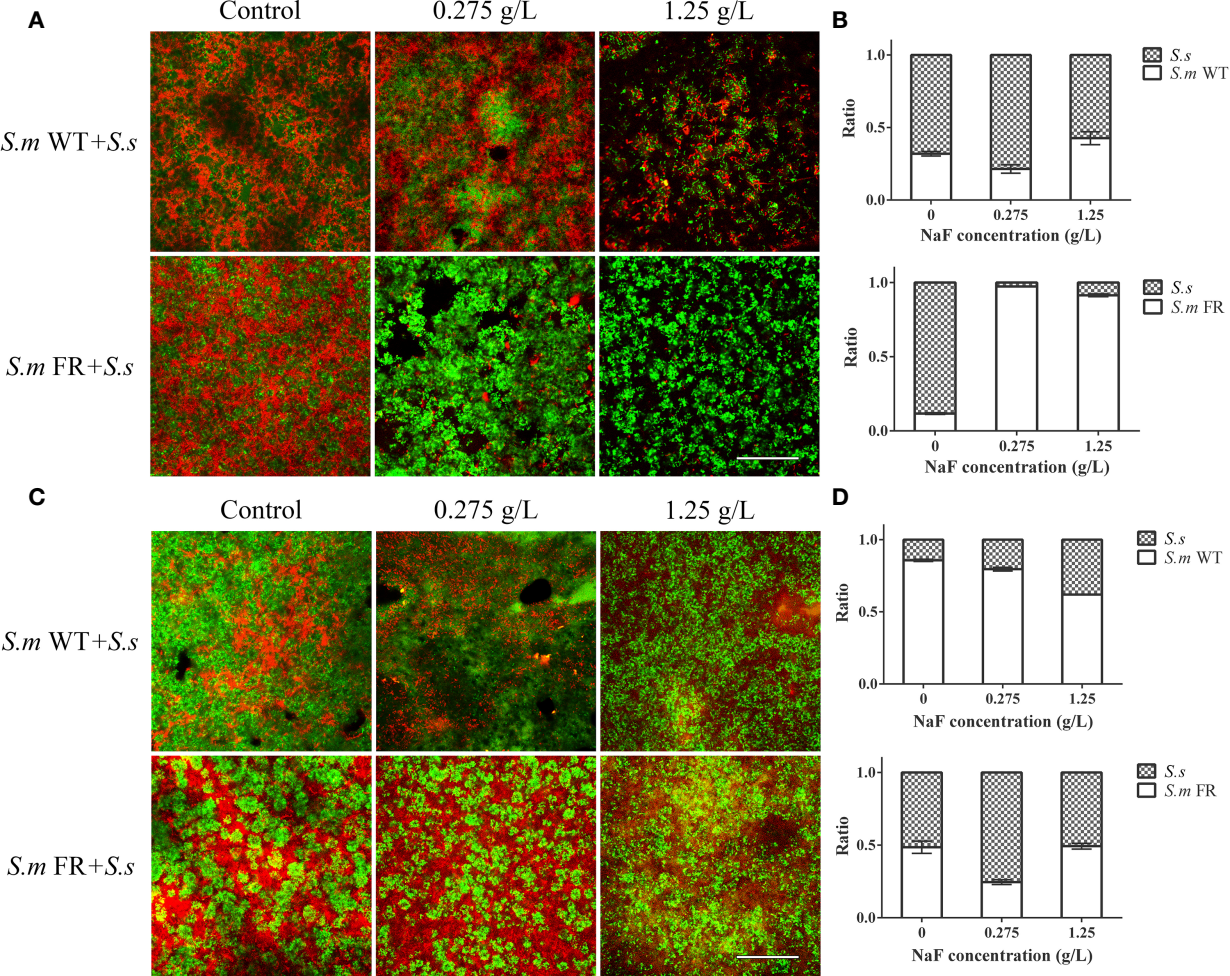
The composition of dual-species biofilm was monitored using fluorescence *in situ* hybridization (FISH) and qRT-PCR. **(A)** FISH images of 24-h dual-species biofilm in bioflm formation. **(B)** Composition of dual-species biofilm based on qRT-PCR in bioflm formation. **(C)** FISH images of pre-formed bioflms. **(D)** Composition of dual-species pre-fromed biofilm based on qRT-PCR. *S. m* was labeled in green, while *S. sanguinis* was in red. Size marker equals 60 μm.

The ratio of *S.m* and *S.s* in dual-species biofilms was analyzed using FISH and qRT-PCR (**Figure 1**). We found that *S.s* had an advantage (more than 50%) in competition with *S.m* WT and FR without fluoride in biofilm formation. In the fluoride-free group, *S.m* FR accounted for 11.72% of dual-species biofilms, while *S.m* WT accounted for 31.93%. However, with the addition of NaF, the proportion of *S.m* FR (over 90%) was much higher than that of *S.s*, occupying a dominant position in dual-species biofilms. However, the ratio of *S.m* WT (less than 50%) maintained a previous trend with the effect of NaF. Surprisingly, in the pre-formed biofilm, *S.m* FR did not gain advantage over *S.s* under NaF-like biofilm formation, and the proportion of *S.m* WT was higher than that of *S.s* in all experimental groups in the pre-formed biofilm.

### NaF Had Different Effects on Biofilm Formation and Pre-formed Biofilm Even in WT Strains

**Figure 2:**
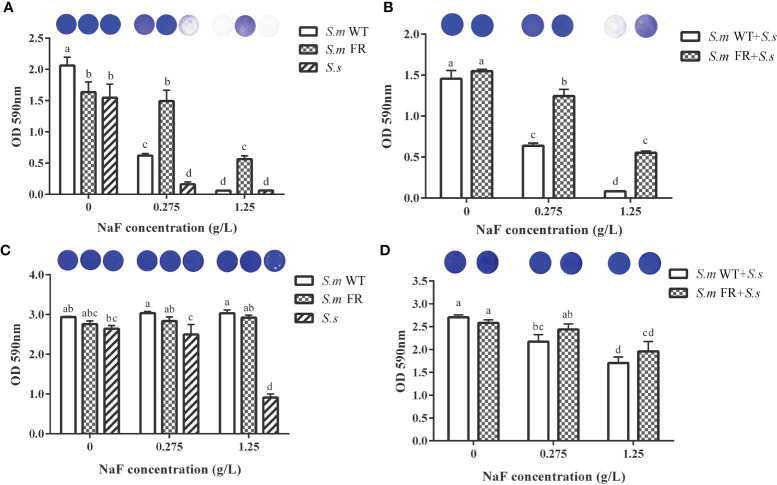
Biomass measured using crystal violet staining. **(A)** Single-species biofilm formation. **(B)** Dual-species biofilm formation. **(C)** Pre-formed single-species biofilms. **(D)** Pre-formed dual-species biofilms. The same letters indicate no statistical difference, and different letters indicate a significant difference between groups (*P* < 0.05).

**Figure 3:**
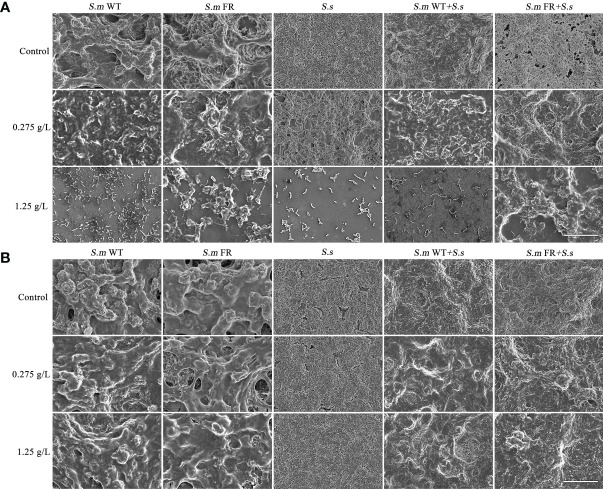
SEM images of the structure of biofilm in different NaF concentrations at ×2,000 magnification. **(A)** Biofilm formation. **(B)** Pre-formed biofilms. Size marker equals 20 μm.

Using CV staining, we compared the biomass of different biofilms under NaF treatment (**Figure 2**). During the biofilm formation of single-species biofilms, *S.m* FR showed a stronger biofilm-forming ability than both *S.m* WT and *S.s* under NaF and even formed a robust biofilm at 1.25 g/L NaF (**Figure 2A**). For the biofilm formation of dual-species, *S.m* FR + *S.s* also showed observably improved biofilm formation capability under NaF (**Figure 2B**). However, NaF had a little anti-biofilm effect on both *S.m* FR and *S.m* WT in pre-formed biofilms (**Figure 2C**). The pre-formed *S.s* biofilms did not show strong resistance as two *S.m* strains under NaF and its biofilm biomass were reduced significantly under 1.25 g/L NaF (**Figure 2C**). In the pre-formed dual-species biofilm, both types of dual-species biofilms withstood NaF stress and only decreased by 19.77 and 36.93% for *S.m* WT + *S.s* and 5.57 and 24.13% for *S.m* FR + *S.s* under 0.275 and 1.25 g/L NaF, respectively (**Figure 2D**). The SEM results also showed a similar tendency in which FR strain-related biofilms acquired survival advantage at 1.25 g/L NaF during biofilm formation, thus forming a more robust biofilm than that of the WT biofilms (**Figure 3**). However, the fluoride resistance advantage of *S.m* FR was not highlighted in the pre-formed biofilms (**Figure 3**).

### Biofilm Formation and Pre-formed Biofilm Showed Different Susceptibilities to NaF in Metabolic Activity Even When *S.ms* Was Compared to its Fluoride-Resistant Strain-Related Biofilms

**Figure 4:**
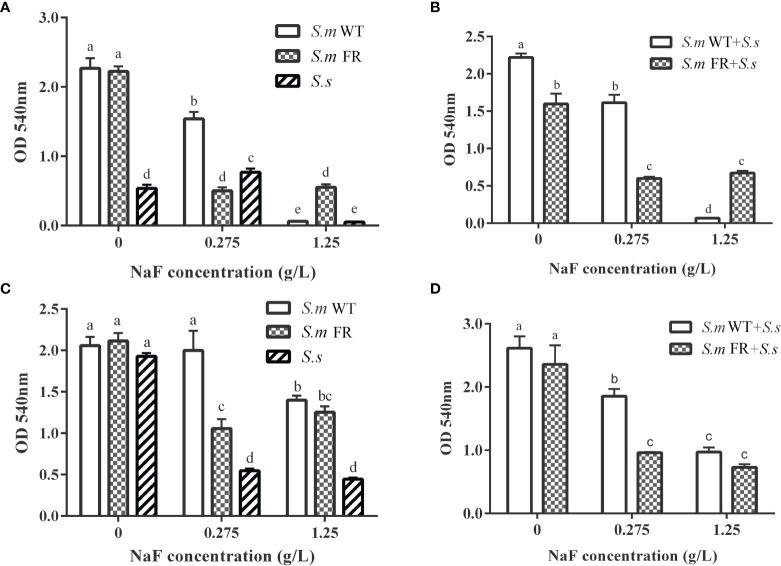
Metabolic activity measured using MTT assay. **(A)** Single-species biofilm formation. **(B)** Dual-species biofilm formation. **(C)** Pre-formed single-species biofilms. **(D)** Pre-formed dual-species biofilms. The same letters indicate no statistical difference, and different letters indicate a significant difference between groups (*P* < 0.05).

The MTT assay was used to detect the metabolic activity of the biofilms (**Figure 4**). In general, NaF suppressed the metabolic activity of biofilms, while this inhibitory effect was different between biofilm formation and pre-formed biofilms. At 0.275 g/L NaF, all *S.m* and its containing groups showed greater metabolic activity than the fluoride-resistant and *S.s* strains. During biofilm formation, either *S.m* FR or *S.m* FR + *S.s* involving dual-species biofilm showed a higher metabolic activity than the other groups under NaF at 1.25 g/L (**Figures 4A, B**). Similar to the CV results in pre-formed biofilms, although NaF showed a suppressive effect on metabolic activity in a strain-, species-, and dose-dependent manner, all pre-formed biofilms could still sustain biofilm even at 1.25 g/L NaF (**Figures 4C, D**). There were no obvious differences between *S.m* WT and *S.m* FR as well as their involved dual-species biofilms at 1.25 g/L NaF (**Figures 4C, D**).

### Fluoride-Resistant *S.m*-Related Biofilms Produce More EPS Than WT Strain Under Fluoride

**Figure 5:**
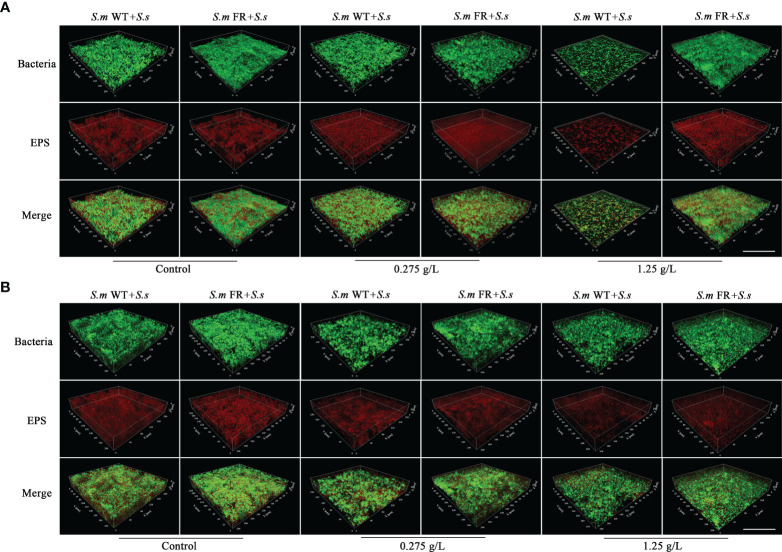
Representative confocal images of EPS staining in different NaF concentrations. **(A)** Dual-species biofilm formation. **(B)** Pre-formed dual-species biofilms. Total bacterial cells were labeled with SYTO 9 (green) and exopolysaccharide with Alexa Fluor 647 (red). Size marker equals 100 μm.

**Figure 6:**
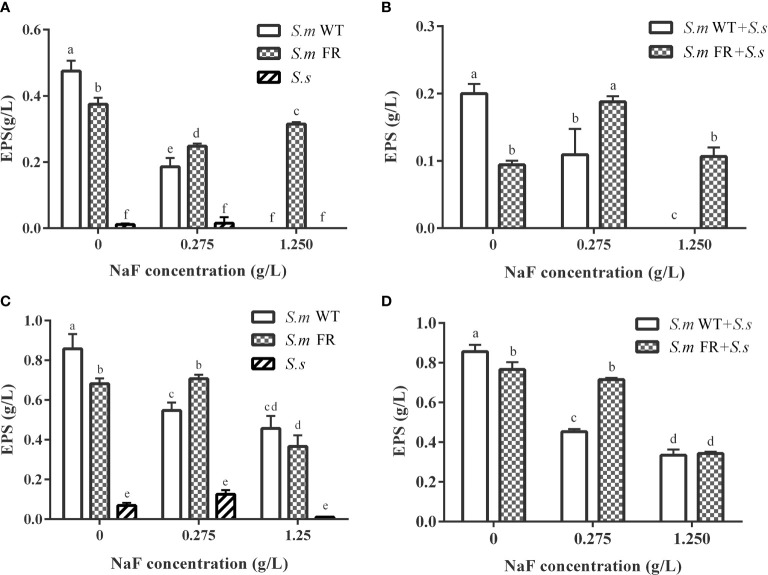
Water-insoluble exopolysaccharide measured using anthrone method. **(A)** Single-species biofilm formation. **(B)** Dual-species biofilm formation. **(C)** Pre-formed single-species biofilms. **(D)** Pre-formed dual-species biofilms. The same letters indicate no statistical difference, and different letters indicate a significant difference between groups (*P* < 0.05).

EPS staining (**Figure 5**) and the anthrone method (**Figure 6**) were used to measure biofilm EPS production. The biofilm formation results showed that fluoride-resistant strain-related biofilms produced less EPS than WT strains without fluoride, while there was more EPS under fluoride (**Figures 5A** and **6A, B**). In pre-formed biofilms, the results were disparate (**Figures 5B** and **6C, D**). Although fluoride-resistant *S.m*-related biofilms synthetized more EPS at 0.275 mg/L, there were no significant differences between fluoride-resistant *S.m*-related biofilms and their WT biofilms at 1.25 g/L (**Figures 6C, D**). Analogous results of EPS production were also confirmed by the SEM images (**Figure 3**).

### Fluoride-Resistant *S.m*-Related Biofilms Had Lower Supernatant pH Than Wild-Type Strains in All NaF-Containing Groups Except in Pre-Formed Biofilms at High NaF

**Figure 7:**
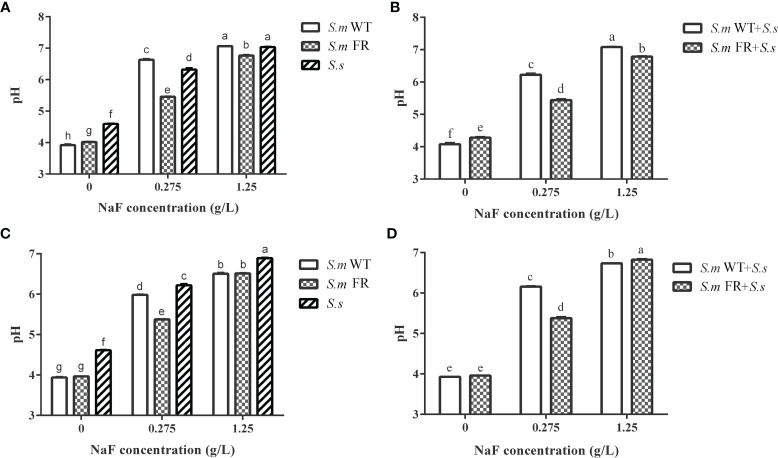
pH of culture supernatant of biofilm. **(A)** Single-species biofilm formation. **(B)** Dual-species biofilm formation. **(C)** Pre-formed single-species biofilms. **(D)** Pre-formed dual-species biofilms. The same letters indicate no statistical difference, and different letters indicate a significant difference between groups (*P* < 0.05).

In total, NaF treatment resulted in a higher pH of the biofilm supernatant (**Figure 7**). During biofilm formation, *S.m* WT and *S.m* WT + *S.s* had a lower pH than *S.m* FR and *S.m* FR + *S.s* without NaF (**Figures 7A, B**). However, the opposite was observed with the addition of NaF, as *S.m* FR and *S.m* FR + *S.s* had a lower pH (**Figures 7A, B**). In pre-formed biofilms, *S.m* FR and *S.m* FR + *S.s* had a lower pH than *S.m* WT and *S.m* WT + *S.s* only at 0.275 mg/L (**Figures 7C, D**).

### Fluoride-Resistant Strain-Related Biofilms Showed Stronger Lactic Acid Production Under High Fluoride Concentration in Biofilm Formation While Not in Pre-Formed Biofilms

**Figure 8:**
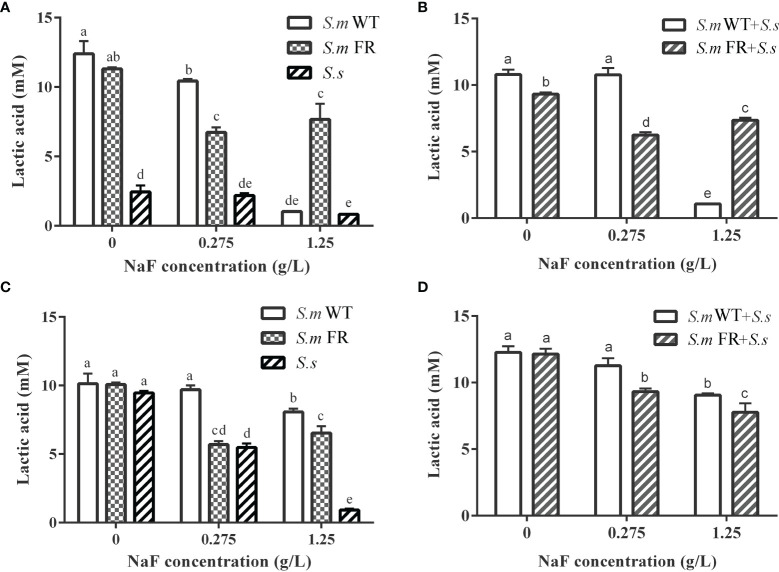
Lactic acid production of biofilms. **(A)** Single-species biofilm formation. **(B)** Dual-species biofilm formation. **(C)** Pre-formed single-species biofilms. **(D)** Pre-formed dual-species biofilms. The same letters indicate no statistical difference, and different letters indicate a significant difference between groups (*P* < 0.05).

Lactic acid production in the two models of biofilms, biofilm formation and pre-formed biofilms, was also detected. The lactic acid measurements showed that the production of lactic acid by fluoride resistance was inhibited by 0.275 g/L (**Figures 8A, B**). At 1.25 g/L, the lactic acid production of *S.m* FR and *S.m* FR + *S.s* was much higher than that of *S.m* WT and *S.m* WT + *S.s* in biofilm formation (**Figures 8A, B**). Surprisingly, in the pre-formed biofilm, *S.m* FR and *S.m* FR + *S.s* produced less lactic acid than *S.m* WT and *S.m* WT + *S.s* under NaF (**Figures 8C, D**).

## Discussion

The present study investigated whether fluoride-resistant *S. mutans* would influence oral microecological homeostasis under fluoride in an antagonistic dual-species biofilm model to further investigate whether fluoride-resistant strains would influence the anti-caries effect of fluoride. A dual-species biofilm composed of *S. mutans* and *S. sanguinis* was chosen, as homeostasis of this dual-species biofilm used in our study could also represent dental plaque balance to a certain degree. Both dual-species biofilm formation and pre-formed biofilm were monitored, and our results showed that fluoride-resistant *S. mutans* influenced the composition, biomass, structure, metabolic activity, acid production, and EPS production of dual-species biofilms when compared with wild-type biofilms under NaF. Fluoride-resistant *S. mutans* had a survival advantage and stronger cariogenic potency in dual-species biofilm formation under NaF but could not highlight its fluoride-resistant superiority thoroughly in pre-formed dual-species biofilms under NaF.

The lower ratio of the fluoride-resistant strain within the dual-species biofilm without NaF compared to its wild strain might be partly attributed to the slow growth rate of the fluoride-resistant strain in our study (data not shown). The slow growth rate of the fluoride-resistant strain was consistent with previous reports, which might have resulted from bacterial-deficient carbohydrate uptake (Liao et al., 2015; Lee et al., 2021). The ratio of either *S. mutans* or its fluoride-resistant dual-species was raised without NaF with the development of biofilm when compared between 24 and 48 h, which was confirmed by FISH and qRT-PCR. This tendency was similar to a previous study on the association between *S. mutans* and *S. sanguinis*. It has been reported that the level of *S. mutans* is lower than that of *S. sanguinis* in the initial biofilm and higher in the mature biofilm (Mitrakul et al., 2016). However, in line with our expectations, the fluoride-resistant *S. mutans* was at an advantage in competition with *S. sanguinis* with the addition of NaF in biofilm formation owing to its fluoride-resistant properties, which could be supported by the biomass and metabolic activity of single-species biofilm formation. Surprisingly, in the pre-formed biofilm, the trend was completely different, as fluoride-resistant *S. mutans* did not achieve a competitive advantage within dual-species biofilms. The different outcomes of fluoride-resistant *S. mutans* within dual-species biofilms between biofilm formation and pre-formed biofilms under NaF might be explained as follows: NaF was added at the beginning of biofilm formation, and its screening effect on bacterioplankton was effective immediately, resulting in a survival advantage of the fluoride-resistant strain. Once dominant in biofilms, *S. mutans* produces more acid and creates an environment conducive to its own growth, thus taking an advantage over *S. sanguinis* (Takahashi and Nyvad, 2011). Nevertheless, in preformed biofilms, NaF was added after 24-h formed biofilms. The biofilms are more resistant to drugs than planktonic bacteria as reported previously (Stewart and Costerton, 2001). Owing to the resistance of mature biofilms to drugs, the survival advantage of the fluoride-resistant strain under NaF was almost entirely covered in the pre-formed biofilm.

Fluoride can influence the adherence of *S. mutans*, a dominant cariogenic virulence (Shani et al., 2000). It has been reported that fluoride-resistant strains retain more adherence ability under fluoride (Men et al., 2016). EPS plays an important role in the stability of biofilms and adhesion to tooth surfaces (Flemming and Wingender, 2010). In biofilm formation, the EPS production of dual-species biofilms of fluoride-resistant *S. mutans* was lower than that of the wild strain without NaF. However, under fluoride, the EPS production of dual-species biofilms containing fluoride-resistant *S. mutans* was significantly higher than that of the wild strain, which was the same as that of the single-species biofilms. In pre-formed biofilms, fluoride-resistant *S. mutans*-related biofilms only produced more EPS at 0.275 g/L NaF. EPS is known to help bacteria increase the resistance of biofilms to escape antibiotic drugs and immune responses (Ðapa et al., 2013). More EPS accumulation may enhance the resistance and adherence of biofilms and even cause higher cariogenicity. This could partly explain why pre-formed biofilms were more resistant to NaF. As a major factor in cariogenicity, glucosyltransferases (Gtfs) play a critical role in EPS formation (Bowen and Koo, 2011). However, previous studies found that there was no inhibition of Gtfs activity of the *S. mutans* wild strain by fluoride (Pandit et al., 2011; Guo et al., 2014). Whether the Gtfs activity of our fluoride-resistant *S. mutans* was suppressed by NaF needs to be further investigated. We were unaware whether there was a direct relationship between fluoride resistance acquisition and EPS production as shown in single-species biofilm results without NaF. The survival advantage of fluoride-resistant *S. mutans* made a significant contribution to the EPS production of its dual-species biofilms during biofilm formation.

Acid production is also an important factor in cariogenic virulence. In general, NaF had an inhibitory effect on acid production in biofilms, whether in single or dual species, according to our data, which was consistent with a previous report (Liao et al., 2017). At present, there is no unified conclusion about the variation in acid production ability of wild-type *S. mutans* compared with fluoride-resistant *S. mutans*. Some studies found that fluoride-resistant *S. mutans* had a weaker acid production ability, while others found it to be stronger when compared to its related wild strain, and further studies found that there was no significant difference between these two strains (Eisenberg et al., 1985; Van Loveren et al., 1991; Hoelscher and Hudson, 1996; Cai et al., 2017; Lee et al., 2021). This diversity might be derived from different culture conditions and bacterial strains and induced by fluoride-resistant strains. In our study, fluoride-resistant *S. mutans*-related biofilms had a lower supernatant pH than the wild-type strains in all NaF-containing groups except in pre-formed biofilms at high NaF, indicating a greater cariogenic potential. This result may partly result from the suppression effect of NaF on acid production. During the lactic acid production process without NaF, fluoride-resistant *S. mutans*-related biofilms showed stronger lactic acid production under a high fluoride concentration in biofilm formation, but not in pre-formed biofilms. Lactic acid production was derived from carbohydrate metabolism (Krzyściak et al., 2014). The lactic acid result may be partly attributed to the stronger resistance to NaF in pre-formed biofilm, including the wild-type ones, resulting in an entirely different trend of biofilm metabolic activity when compared to that of biofilm formation, which would further influence lactic acid production. In addition, biofilm composition also contributed to the observed difference, as *S. sanguinis* produced less acid than *S. mutans* and its fluoride-resistant strain within the biofilm. The inconformity between pH and lactic acid results originated from the methods used. For lactic acid measurement, after 24 h of biofilm formation or treatment of pre-formed biofilm with NaF for another 24 h, the resulting biofilms were used for lactic acid production without fluoride. For pH, the culture medium contained fluoride for 24 h in both the biofilm formation and pre-formed biofilms. We hypothesize that the acidogenicity of the fluoride-resistant strain in our study was higher than that of wild strains under fluoride in biofilm formation, which is consistent with previous studies of single-species biofilms (Van Loveren et al., 1991; Hoelscher and Hudson, 1996; Sheng and Liu, 2000). However, this preponderance could not be observed in the pre-formed biofilms.

Although the balance between *S. mutans* and *S. sanguinis* could represent the dental plaque equilibrate to some extent, dental plaque is intricate (Sun et al., 2019). Further studies need to be conducted using saliva or *in vivo* biofilms to evaluate the impact of fluoride-resistant strains on the micro-ecology of dental plaque. In addition, studies on the use of more clinically isolated fluoride-resistant strains, including *S. mutans*, were encouraged, as lab-induced fluoride-resistant strains might provide different results from clinical isolates. There is no doubt that a comprehensive understanding of the fluoride-resistant mechanism would inspire more methods to control these fluoride-resistant opportunistic cariogenic bacteria. Drug resistance is a worldwide crisis, especially in the post-antibiotic era. To inhibit biofilm formation containing drug-resistant bacteria, controlling drug-resistant strains should be considered; otherwise, it would occupy an absolute ecological advantage, as in our study. The pre-formed biofilms were more resistant than biofilms during formation in our study, just as reported before (Angelopoulou et al., 2020). Dispersal molecules might be a route to consider, which could trigger biofilm degradation and disperse pre-formed biofilms to the bacterioplankton state and thus could control it by inhibiting biofilm formation (Fleming and Rumbaugh, 2017). Moreover, although fluoride had less impact on controlling fluoride-resistant *S. mutans* biofilms, especially in biofilm formation, whether fluoride-resistant *S. mutans* would finally disrupt homeostasis between demineralization and remineralization remains to be further studied. Fluoride could inhibit demineralization and enhance remineralization, with the exception of the antibacterial effect.

In summary, this study investigated the effect of fluoride-resistant *S. mutans* on microecological homeostasis using an antagonistic dual-species biofilm model under fluoride. Under the screening effect of fluoride, fluoride-resistant *S. mutans* gained a survival advantage within antagonistic dual-species biofilms during biofilm formation, thus disrupting the ecological balance. Fluoride-resistant *S. mutans* also exhibited stronger cariogenic virulence, including acidogenicity and EPS production, which might further influence the anti-caries effect of fluoride from the perspective of biofilm control. However, in pre-formed biofilms, even wild-type *S. mutans* containing dual-species biofilms showed strong resistance, and the advantage of fluoride-resistant *S. mutans* could not be fully highlighted for biofilm formation. However, this does not mean that fluoride is invalid for fluoride-resistant strains. Inhibition of biofilm biomass, metabolism, acidogenicity, and EPS production was found within biofilms under NaF.

## Data Availability Statement

The original contributions presented in the study are included in the article/**Supplementary Material**. Further inquiries can be directed to the corresponding authors.

## Author Contributions

QY and YH designed this project. KZ and YX conducted experiments and acquired the data. YP, FT, YX, YC, and ZX analyzed and interpreted the data. QY, YH, YL, and XL polished the language. KZ and YX wrote the main manuscript text. KZ and YS acquired funding. All authors contributed to the article and approved the submitted version.

## Funding

This study was supported by the National Natural Science Foundation of China (grant no. 82001041), Zhejiang Provincial Natural Science Foundation of China (grant no. LGF20H140001 and LGF19H140004), and Wenzhou Technology Bureau Project (grant no. Y20190487).

## Conflict of Interest

The authors declare that the research was conducted in the absence of any commercial or financial relationships that could be construed as a potential conflict of interest.

## Publisher’s Note

All claims expressed in this article are solely those of the authors and do not necessarily represent those of their affiliated organizations, or those of the publisher, the editors and the reviewers. Any product that may be evaluated in this article, or claim that may be made by its manufacturer, is not guaranteed or endorsed by the publisher.
