# Supplementary material for: Influence of Fluoride-Resistant Streptococcus mutans Within Antagonistic Dual-Species Biofilms Under Fluoride In Vitro
Source: Front Cell Infect Microbiol. 2022 Feb 28;12:801569. doi: 10.3389/fcimb.2022.801569 (PMC8918626; doi:10.3389/fcimb.2022.801569)
Supplement: Supplementary file 1 [file Table_1.docx]

Supplementary Material

**Supplementary Table 1.** The primers of *S. mutans* and *S. sanguinis* used in real-time quantitative PCR assay.

| **primer** | | **Nucelotide Sequence (5’-3’)** | **Reference** |
| --- | --- | --- | --- |
| **S. mutans** | Forward | 5’-AGTCGTGTTGGTTCAACGGA-3’ | (Huang et al., 2015) |
|  | Reverse | 5’-TAAACCGGGAGCTTGATCGG-3’ | (Huang et al., 2015) |
| **S. sanguinis** | Forward | 5’-TCAGCAAATCCCCCAGGTTC-3’ | (Huang et al., 2015) |
|  | Reverse | 5’-AACGGAGTGTCAGCGAAGTT-3’ | (Huang et al., 2015) |

**Supplementary Table 2.** Oligonucleotide primers of *S. mutans* and *S. sanguinis* used in fluorescent in situ hybridization (FISH).

| **Probes** | **Nucelotide Sequence (5’-3’)** | **Reference** |
| --- | --- | --- |
| **Streptococcus. mutans** | Alexa Fluor 488-5’-ACTCCAGACTTTCCTGAC-3’ | (Zheng et al., 2013) |
| **Streptococcus. sanguinis** | Alex Fluor 594-5’-GCATACTATGGTTAAGCCACAGCC-3’ | (Zheng et al., 2013) |

**Reference**

Huang, R., Zhang, J., Yang, X.F., and Gregory, R.L. (2015). PCR-Based Multiple Species Cell Counting for In Vitro Mixed Culture. *PLoS One* 10**,** e0126628.

Zheng, X., Zhang, K., Zhou, X., Liu, C., Li, M., Li, Y., Wang, R., Li, Y., Li, J., Shi, W., and Xu, X. (2013). Involvement of gshAB in the interspecies competition within oral biofilm. *J Dent Res* 92**,** 819-824.
